# Recurrent Respiratory Papillomatosis (RRP)—Meta-analyses on the use of the HPV vaccine as adjuvant therapy

**DOI:** 10.1038/s41541-023-00644-8

**Published:** 2023-04-01

**Authors:** Peter Goon, Odile Sauzet, Matthias Schuermann, Felix Oppel, SenYao Shao, Lars-Uwe Scholtz, Holger Sudhoff, Martin Goerner

**Affiliations:** 1 Department of Medicine, Yong Loo Lin School of Medicine, National University of Singapore, and Departments of Dermatology & Otolaryngology, National University Health System, Singapore, Singapore; 2 Bielefeld School of Public Health and Department of Business Administration and Economics, University of Bielefeld, Universitätsstraße 25, 33615 Bielefeld, Germany; 3 University Dept of Otolaryngology, Campus Klinikum Bielefeld Mitte, University Hospital OWL of Bielefeld University, Teutoburger Str. 50, 33604 Bielefeld, Germany; 4 Dept of Haematology, Oncology and Palliative Medicine, Campus Klinikum Bielefeld Mitte, University Hospital OWL of Bielefeld University, Teutoburger Str. 50, 33604 Bielefeld, Germany

**Keywords:** Tumour virus infections, Viral infection

## Abstract

Recurrent Respiratory Papillomatosis(RRP) is a rare disease with severe morbidity. Treatment is surgical. Prevailing viewpoint is that prophylactic HPV vaccines do not have therapeutic benefit due to their *modus operandi*. Studies on HPV vaccination alongside surgery were meta-analysed to test effect on burden of disease. Databases were accessed Nov and Dec 2021 [PubMed, Cochrane, Embase and Web of Science]. Main outcome measured was: Mean paired differences in the number of surgeries or recurrences per month. Analyses was performed using: Random effect maximal likelihood estimation model using the Stata module Mataan(StataCorp. 2019. *Stata Statistical Software: Release 16*. College Station, TX:StataCorp LLC.) Our results found *n* = 38 patients, suitable for syntheses with one previous meta-analyses (4 published, 2 unpublished studies) *n* = 63, total of *n* = 101 patients. Analyses rendered an overall reduction of 0.123 recurrences or surgeries per month (95% confidence interval [0.064, 0.183]). Our meta-analyses concludes that HPV vaccine is a beneficial adjunct therapy alongside surgery

## Introduction

Recurrent Respiratory Papillomatosis is a rare disease that can have severe morbidity, due to recurrent growth of papillomata that threaten to obstruct the airways. Its aetiology has been clearly defined, and over 90% of cases are thought to be due to Human Papillomavirus (HPV) types 6 and 11^1,2^, which are also the dominant types causing ano-genital warts, but high risk subtypes have also been identified^3–5^. The morbidity results from numerous repeated surgeries and can cause scarring of the vocal cords resulting in hoarseness and/or change in voice generation.

The development of the HPV prophylactic vaccines has been a major advance in the field. There is currently a nonavalent vaccine (Gardasil 9—licensed 2014) in use in the US, and the UK is due to change from Gardasil (tetravalent vaccine since 2012) to Gardasil 9 for its national HPV vaccination programme imminently. Safety profiles and efficacy rates in infection prevention have been similarly excellent. Recent UK data reporting on the national HPV vaccination programme^6^ have now demonstrated the efficacy of these vaccines to prevent the development of cervical cancer at the population level. Australia has reported on the impressive reduction of RRP within their highly vaccinated population^7^, and preliminary data from the US show a similar trend^8^.

The age at onset of RRP was previously thought to possess a bimodal distribution with juvenile onset (JORRP) and adult onset (AORRP) peaks at approximately 5 and 30 years respectively (Cohn et al. has been recurrently cited but does not actually give any evidence for those assumptions^9^), but a study in 2015 from 12 European hospitals^10^ with *n* = 639, has now demonstrated that there is a trimodal distribution with peaks at median ages of 7, 35 and 64 years of age^10^.

Reported incidence rates for JORRP and AORRP are estimated at 0.17–1.34 per 100,000 and 0.18–0.54 per 100,000 respectively^11–15^. In the adult population, the 2 peaks appear to correlate to the oral cavity HPV infection prevalences as reported by Gillison et al. in 2012^16^. Adult HPV transmission appear mainly to be via intimate contact. Gender distribution in adults appear to follow that of HPV oral infection also (2–3 males to 1 female). JORRP appear to be predominantly via vertical transmission (mother to child during labour and vaginal delivery) with a 200-fold increase in risk for mothers with a history of genital wart infection^17^. However, elective caesarean section to prevent vertical transmission is controversial and has not been shown to definitively reduce transmission^18,19^. Early age of presentation with papillomatosis in a child (<3 years) appears to be a risk factor for severe disease and increased recurrences^20^. Interestingly, infection with the HPV 11 type appears to be associated with more severe disease, more recurrence, distal spread into the larynx, and even malignant transformation^20–22^. This is in contra-distinction with HPV 6, which is the dominant type found in ano-genital disease.

HPV are small (~8 kb) non-enveloped viruses, but have an icosahedral protein capsid formed out of 72 L1 (with monomeric L2 protein embedded within the centre) pentamers. Electron microscopy measurements estimate a virion size of 52–55 nm, and there are 8–10 genes (median of 8) encoded by a double stranded DNA genome. HPV tend to produce papillomata at the transformation zones (where there is transition between squamous and columnar epithelia) but can infect anywhere in the aero-digestive tract or ano-genital mucosa or skin anywhere on the body. L1 protein was found to self-assemble into their native conformation (Jian Zhao et al.^23^ in 1991), and proved highly immunogenic in producing neutralizing antibodies. These discoveries have proved to be the basis of the highly successful L1 vaccines produced in bivalent (Cervarix™), quadrivalent (Gardasil™) and nonavalent (Gardasil 9™) forms and which have been shown to reduce incidence rates of cervical cancer (and dysplastic precursors)^6^ and also RRP^7^ in current real world experience.

There are numerous other L1 vaccine clinical trials ongoing:^24^ to test the efficacy of a 1 dose regimen; efficacy of 3 doses in 20–45 year old females; usage in postpartum women and immunocompromised individuals (HIV and transplant patients); dosage sparing via intradermal injections; usage as therapy in combination with a PD1 inhibitor in patients with cervical cancer; efficacy in protecting against recurrent disease in women undergoing ablative therapy for cervical disease, persistence of protection and efficacy in preventing oropharyngeal cancers.

**Fig. 1:**
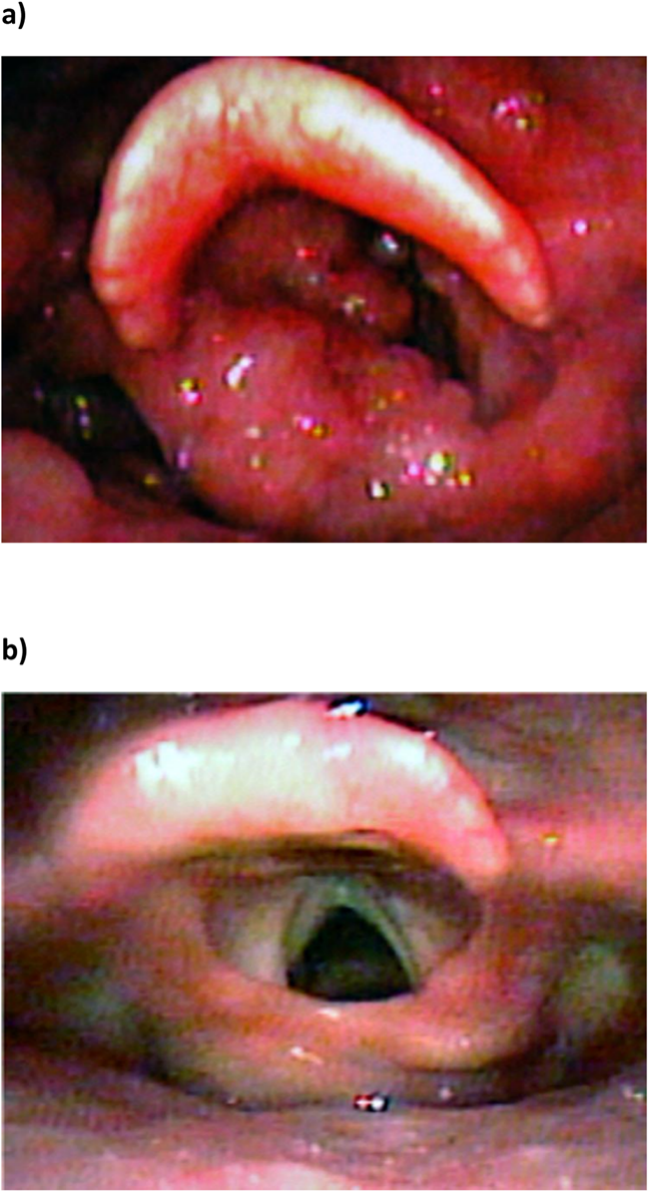
Juvenile Recurrent Respiratory Papillomatosis. **a** At Surgery (>80 surgeries in previous 4 years). **b** Three months Post-Surgery (1a) and HPV vaccine.

L1 HPV vaccine has also been used as adjunct therapy in the treatment of RRP. The evidence of efficacy in reducing recurrent papillomata formation, reducing the number of surgeries to keep airways patent, increasing the interval between surgeries, etc. have been accumulating gradually over the years^24^. Our group as well as others has published on this interesting observation^25^ (see Fig. 1). The majority of published data on HPV vaccine as adjunct in RRP have been conducted in small cohort single-centre studies, since RRP is a rare disease. Despite the clinical evidence so far, the prevailing dominant viewpoint is that L1 vaccine cannot give therapeutic benefit since its primary function is to generate protection against infection via anti-L1 antibodies (its *modus operandi*).

There was a meta-analyses published in 2019 by Rosenberg et al.^26^ which demonstrated beneficial effects of the quadrivalent HPV vaccine, in conjunction with surgery for RRP. Synthesis analyses from 4 published and 2 unpublished datasets yielded 63 patients suitable for meta-analyses at that time. Mean intersurgery intervals (ISIs) improved from 7.02 months (range 0.30–45.0 months) prior to vaccination, to 34.45 months (range 2.71–82.0) post-vaccination. Here we have updated and supplemented the meta-analyses with patients from new studies we were able to find since 2018 to gather the available evidence that HPV vaccine can be used to substantial beneficial effect for RRP (as adjunct treatment alongside surgery). An updated meta-analyses would be most useful in rapid dissemination of this information, since there are likely to be 28,000–150,400 patients (assuming a world population of 8 billion) estimated to be living and suffering with RRP all across the world.

## Results

### Results from search process and mathematical synthesis

**Fig. 2:**
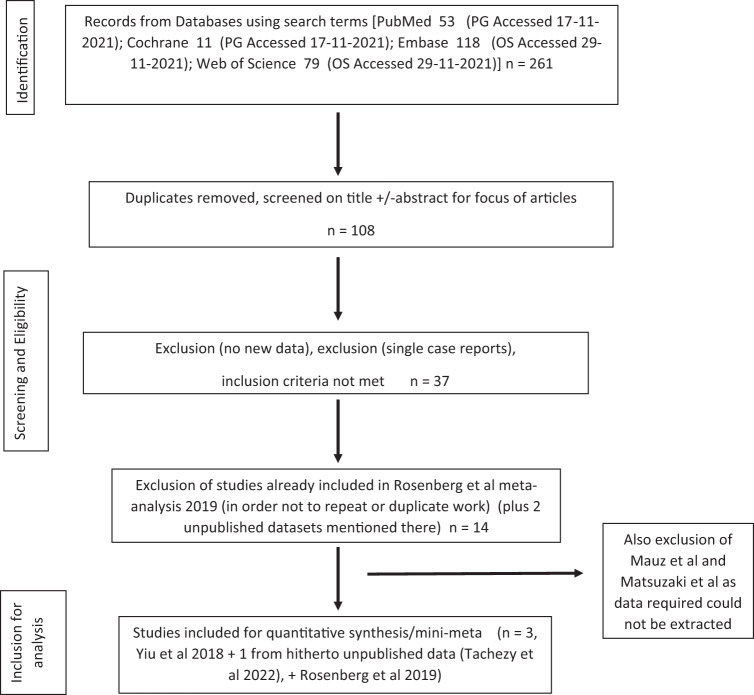
Flow diagram for search. PRISMA flow diagram.

Figure 2 illustrates the literature search process. After undergoing this rigorous selection process, (and including the previous published data from Rosenberg et al.^26^), we found several studies which were potentially suitable for synthesis analyses. Mauz et al. 2018^27^ and Matsuzaki et al. 2019^28^ were then excluded due to the lack of pre-immunisation data which was required for analyses. Personal communications with Smahelova et al. 2022^29^ allowed us access to unpublished and just published data and has helped to reduce bias. See Table 1 for details of the patients included in the analyses.

**Table 1:** HPV-vaccinated patients included in meta-analysis.

	JORRP: AORRP	Pts who received other Adjuvants	Median Age (range)	Sex (M: F)	Race/ethnicity	Comments
Yiu et al.	4: 38 but all 12 patients included appear to be AORRP	6 (3—Cidofovir alone), 3—Cidofovir and Bevacizumab	46 years (34–56)	10: 4	US study but not mentioned	2 pts excluded due to lack of follow up
Smehalova et al.	Ratio not mentioned but likely most AORRP	None mentioned	Men 44 (21–73) Women 39 (37–48)	39: 11 (total cohort)	Not mentioned but likely central/east European	26 pts included
Rosenberg et al.	Approx 20: 38 (not mentioned in 1 study)	NA	NA	Approx 41: 27	NA but all studies were European	63 pts included by Rosenberg et al.

**Fig. 3:**
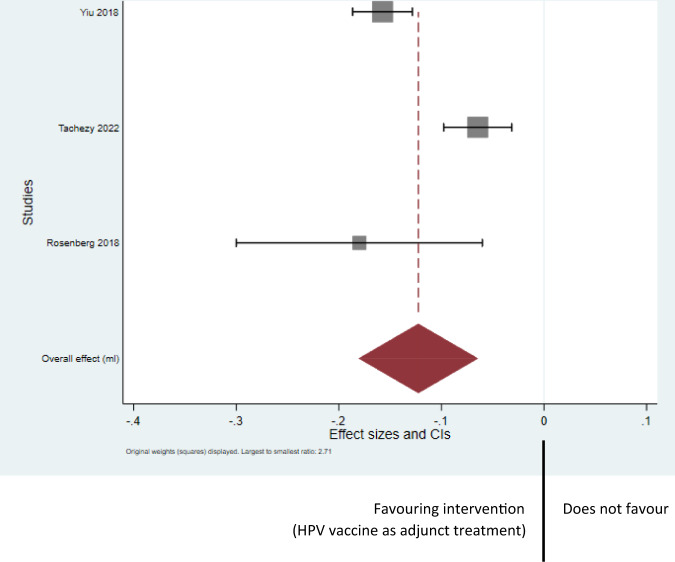
Analysis - Forest Plot of Random Effect Maximal Likelihood Estimation Model. Forest plot of random effect maximal likelihood estimation.

See Fig. 3 for Forest Plot of Random Effect Maximal Likelihood Estimation Model analysis.

Total number of patients included in the analysis was *n* = 12 (Yiu et al. 2018) + 26 (Smahelova et al. 2022) + 63 (Rosenberg et al. 2019) which is *n* = 101 patients. This equates to an overall reduction of 0.123 recurrences or surgeries per month (95% confidence interval [0.064, 0.183]).

For the Yiu et al. 2018 study, the 12 patients included had a mean recurrence of 0.225 per month (SD 0.221) before vaccination and 0.068 (SD 0.072) after vaccination. For Smahelova et al. 2022, this mean recurrence value for the 26 patients included was 0.098 (SD 0.082) before and 0.028 (SD 0.032) after vaccination. We were able to synthesise these results with those of Rosenberg et al. 2019.

Our analyses suggest that there is substantial benefit for the intervention of HPV vaccination used as adjunct therapy in conjunction with surgery and strengthen the conclusions of the meta-analysis performed by Rosenberg et al. in 2019^26^ by providing an updated estimate with increased precision.

## Discussion

Our results support the use of HPV prophylactic vaccination in conjunction with ablative surgery to remove the papillomata, and when more recent studies are combined with the large meta-analysis conducted by Rosenberg et al. 2019, the data lend weight to the argument favouring this intervention for all patients.

Further, the mean recurrences for Smahelova et al. 2022 before immunisation might be a little underestimated, due to (1) a less precisely recorded duration of illness (in whole years). Recording duration of illness in whole years prior to vaccination may mean that it was less precise due to rounding up to a whole number, rather than a precisely recorded timespan in months or days. (2) Their data on mean number of recurrences before immunisation is also much lower than in the other studies, which may explain the smaller effect of immunisation. Vaccination appears to reduce the mean number of recurrences to close to zero or low numbers, therefore a smaller average number of recurrences before immunization means that the decline of the decrease line to the average number of recurrences (a low number) after vaccination is not as steep compared to a population with a high average number of recurrences decreasing to a low number.

This latest meta-analysis has now calculated the effect of HPV prophylactic vaccination on the mean RRP recurrences seen before and after vaccination for *n* = 101 patients. This is the largest number of patients meta-analysed from multiple centres so far, and represents a sum total of published and known unpublished data on this therapeutic option. Taken together, the total available evidence strongly suggests there is a substantial benefit for the use of adjuvant HPV vaccine in conjunction with surgery.

The risks and side-effect profiles of these HPV vaccines are well known and their safety excellent. The US Vaccine Adverse Events Reporting System (VAERS) has reported and concluded that the vast majority (97.4%) of reported events were non-serious, with syncope and vaccine administration errors the commonest problems despite the millions of doses administered^30^, and that the safety profile of the HPV9 vaccine is consistent with its predecessor HPV4 vaccine. The main benefits of the vaccine would be its proven efficacy in prophylaxis against HPV infection, and thus protection against ano-genital and head and neck cancers, (even if RRP continues to recur). The observations that there are some *therapeutic benefits* in reducing recurrence of RRP in patients requires quantification, hence this meta-analyses.

The use of HPV vaccine for therapy remains controversial, as the immune mechanisms that would explain this observed phenomenon is uncertain. HPV vaccines are derived from L1 proteins which have self-assembled into virion-like particles (VLPs) and are highly immunogenic but obviously do not contain any HPV DNA so are unable to replicate or initiate infection. The other primary components are the adjuvant (amorphous aluminium hydroxyphosphate sulphate—AAHS), polysorbate 80 (emulsifier) and yeast proteins (HPV L1 grown in yeast cells to produce industrial quantities of proteins).

The primary function of these vaccines are to produce neutralizing antibody responses against L1 protein, this means that there are high-affinity antibodies generated to bind avidly to L1 protein and neutralise the virus inoculum when contact with the virus occurs. This effect has been shown to be highly effective and long-lasting, and the immune memory generated also leads to an anamnestic response whenever L1 is encountered in the future. Real world data on reduction of cervical cancers and RRP have now been published (as previously mentioned).

Adjuvants included in vaccines are many-decades old compounds shown to augment and increase the immune response to the target, choice of adjuvant can help to increase or bias the immune response to a specific pathway i.e. Th1 or Th2, innate immunity and NK cell function, etc. The ideal adjuvant would increase or ramp up the specificity and amplitude of the desired immune response. Aluminium salts such as AAHS have been shown to increase the immune response via the T helper (Th2 > Th1) pathways and the innate immune system especially through the DAMPs (Damage-Associated Molecular Patterns) “Danger signals theory” and therefore with downstream activation of the adaptive immune system via Dendritic cells (DCs) and macrophages^31–33^. It is important to remember that aluminium adjuvants also carry a risk of adverse effects but have been administered in a multitude of vaccines (HepA, HepB, Pneumovax, Diphtheria-Tetanus vaccine formulations, BCG, Anthrax, Yellow Fever, etc.) to billions of human beings and their safety has been monitored for many decades.

The arguments for an adaptive immune response after vaccination with HPV L1 vaccines are two-fold. One, the above use of adjuvant tends to cause a wider and stronger immune response with involvement of both innate and adaptive (both humoral and T-cell activation) immunity to L1 initially, but possibly with involvement of other targets through epitope spreading and affinity maturation later on. Two, we need to remember that HPV 6 and 11 cause primarily productive infections, with full-cycle replication of the viral genome and live virus production. This is in contra-distinction to HPV 16 and 18 (plus other high risk subtypes) which cause productive infections initially but eventually integrate into the cellular genome and cause neoplasia in patients with long-term infections (which were not cleared). This tendency to continuously produce live virus in RRP ensures that viral proteins other than L1 are exposed to the immune response, and can be expected to cause a wide and strong immune response to the pathogenic HPV type since there has been activation of both the innate and adaptive arms of the immune system.

The evidence for a beneficial effect in RRP has been accruing slowly over the years since the quadrivalent HPV L1 was licensed in 2006. The lack of therapeutic effect in cervical cancer and its precursors was noted in 2007^34^. Use of the quadrivalent L1 vaccine in RRP patients was initially for its predicted prophylactic effect (which has now been confirmed with reduction of incidence rates at population levels) but it is likely that the beneficial therapeutic effect was noticed shortly after more RRP patients began to get vaccinated.

The most definitive and accepted method of confirming this effect is to conduct a multi-centre randomised double-blinded and placebo-controlled trial with the placebo arm switching over to the HPV vaccine arm after 1^st^ recurrence of papillomata, or after an accepted period of time of 3–6 months (whichever is first). Conducting such an RCT is likely to be prohibitive in cost and time, and so far, has not been done. Here in this updated perspective on HPV viral immunology and vaccinology, we have presented the virological and immunological logic behind the observed therapeutic effect, and updated the meta-analyses to include the latest studies (including unpublished) with available and synthesizable data to give increased precision.

This latest tranche of evidence is to provide clinicians in the field, with the most up-to-date knowledge on the benefits versus risks of using HPV vaccine as an adjunct treatment, and therefore for consideration of the inclusion of HPV vaccine as adjunct therapy alongside surgery in national and international guidelines. Implementation of such clinical practice change is therefore likely only in the richer countries of the world to begin with, but implementation of single dose regimes in lower resource countries should be considered.

## Methods

### Systematic literature review and meta-analysis performed with PRISMA guidelines

Ethical approval not required. A Population, Intervention, Comparison, Outcome and Study design (PICOS) strategy was undertaken, and patients with RRP looked for. HPV vaccination as adjunct therapy was the intervention searched for. Outcomes were the numbers of recurrences or surgeries before and after HPV vaccination, calculated to the mean recurrences per month. We have taken into consideration the natural history of RRP which tends to a reduction in recurrences and surgeries over time, especially in the young. Therefore, results will be expressed as mean reductions per month, since this is easier to calculate for the shorter pre-vaccine periods seen in young children. We excluded case reports or studies with less than 5 patients as anecdotal single case reports have a high risk of assessment and publication bias. The search terms with inclusion and exclusion criteria are included here.

**Search terms** = (HPV vaccine OR human papillomavirus vaccine OR papillomavirus vaccines OR alphapapillomavirus vaccines) AND (HPV OR human papillomavirus OR alphapapillomavirus OR papillomaviridae OR viral warts OR wart virus) AND (RRP OR recurrent papillomatosis OR recurrent respiratory papillomatosis) NOT (Cervical OR Cervical cancer OR Cervix) NOT (head and neck cancer OR HNSCC OR squamous cell carcinoma) NOT (AIN OR Anal Intraepithelial Neoplasia OR Anus OR Anal cancer)

Databases searched with the above highly focused search terminology (and Boolean filters). We have also searched through ClinicalTrials.gov for studies which were not included in the Rosenberg meta-analyses. We found one study for which no published data was available at the time (now just published), and we contacted the authors who kindly provided their data (Smahelova et al. 2022^29^—NCT01375868).

### Screening and checking for eligibility

PG searched and assessed studies for inclusion and exclusion criteria, with help from OS. Duplicates removed, then study abstracts and titles were checked through. All non-studies, conference reports, case reports with <5 patients, reviews without new or relevant data, all non-English studies or reports (Swedish, Danish, German, Norwegian, Chinese, Russian, Dutch all removed).

We included only studies for which it was possible to calculate paired differences in the number of surgery or recurrences per month. We excluded study participants for which no pre-immunisation data were available or if patients were directly immunised at clinical diagnosis. This meant that we were able to pool the data of Yiu et al. 2018, with data from Smahelova et al. 2022 for synthesis with the summarised effects obtained by Rosenberg et al. 2019.

### Synthesis of results

Analyses were performed using Stata (StataCorp. 2019. *Stata Statistical Software: Release 16*. College Station, TX: StataCorp LLC.). We used a random effect maximal likelihood estimation model using the Stata module Mataan^35^.

Majority of patients from the different studies were adults, although the meta-analyses and syntheses will accord equal weighting to both adults and children (<18 year old). Therefore results will tend to represent adults more than children.

### Risk of bias assessment

PICOS and SIGN checklists completed. There is heterogeneity likely due to the following limiting factors for analyses (numerous studies, different methodologies, different parameters of data collected and presented, limited numbers of patients (as all were single centre studies), retrospective data collection). HPV vaccine management in the cohort of synthesised patients were predominantly from HPV4 (quadrivalent vaccine) since the studies were undertaken primarily after the introduction of HPV4 in 2006, whilst HPV9 was licensed in 2014.

Meta-analyses statistical methods are recognized for synthesizing different studies with different parameters which can cause significant heterogeneity such as different surgical techniques, concomitant drug administration, even criteria for initiating surgery. A past history of different therapies was not an exclusion criterion. Different surgeons, different centres, different criteria and techniques for surgery were accepted as significant but unavoidable contributors to increased heterogeneity and risk of bias.

### Patient and public involvement

Our RRP patients and the public were initially involved as we planned the interventional study in 2013 which was published in 2017^25^. The research questions for that study were developed with their benefit, wellbeing, preferences and clinical experience paramount in our minds. This follow-up study performed meta-analyses and synthesis of available published and unpublished data so was more distant from the patient experience. The parents of the child in Fig. 1 gave informed signed consent for publication of the photos, and were closely involved and actively encouraged the publication of our study for as wide dissemination as possible, since they feel that it is really important to publicise the availability of a beneficial adjunct therapy, now that they have seen the benefit that it has given their child after a torrid time with repeat surgeries over the last 4 years. The child remains clear of regrowth 12 months after the last surgery.

### Reporting summary

Further information on research design is available in the Nature Research Reporting Summary linked to this article.

## Supplementary information

REPORTING SUMMARY

## Data Availability

All de-identified data collected for the study will be made available to others, including related documents, available with publication. A copy of the paper with any supplementary data will be available from the archive of the University of Bielefeld Library (Universität Bielefeld Bibliothek) where the data will be made available (publikationsdienste.ub@uni-bielefeld.de); data will be shared with other investigator groups (universities or research councils or charities), some additional restrictions or criteria may apply.
