# Supplementary material for: Recurrent Respiratory Papillomatosis (RRP)—Meta-analyses on the use of the HPV vaccine as adjuvant therapy
Source: NPJ Vaccines. 2023 Apr 1;8:49. doi: 10.1038/s41541-023-00644-8 (PMC10067830; doi:10.1038/s41541-023-00644-8)
Supplement: Supplementary file 1 — REPORTING SUMMARY [file 41541_2023_644_MOESM1_ESM.pdf]

nature portfolio

Corresponding author(s): Peter Goon

Last updated by author(s): Jan 30, 2023

Reporting Summary

Nature Portfolio wishes to improve the reproducibility of the work that we publish. This form provides structure for consistency and transparency in reporting. For further information on Nature Portfolio policies, see our Editorial Policies and the Editorial Policy Checklist.

Statistics

For all statistical analyses, confirm that the following items are present in the figure legend, table legend, main text, or Methods section.

n/a	Confirmed	
☐	☒	The exact sample size (*n*) for each experimental group/condition, given as a discrete number and unit of measurement
☐	☒	A statement on whether measurements were taken from distinct samples or whether the same sample was measured repeatedly
☐	☒	The statistical test(s) used AND whether they are one- or two-sided<br>*Only common tests should be described solely by name; describe more complex techniques in the Methods section.*
☒	☐	A description of all covariates tested
☒	☐	A description of any assumptions or corrections, such as tests of normality and adjustment for multiple comparisons
☐	☒	A full description of the statistical parameters including central tendency (e.g. means) or other basic estimates (e.g. regression coefficient) AND variation (e.g. standard deviation) or associated estimates of uncertainty (e.g. confidence intervals)
☒	☐	For null hypothesis testing, the test statistic (e.g. *F*, *t*, *r*) with confidence intervals, effect sizes, degrees of freedom and *P* value noted<br>*Give P values as exact values whenever suitable.*
☒	☐	For Bayesian analysis, information on the choice of priors and Markov chain Monte Carlo settings
☒	☐	For hierarchical and complex designs, identification of the appropriate level for tests and full reporting of outcomes
☐	☒	Estimates of effect sizes (e.g. Cohen's *d*, Pearson's *r*), indicating how they were calculated

Our web collection on statistics for biologists contains articles on many of the points above.

Software and code

Policy information about availability of computer code

Data collection	No software was used
Data analysis	Stata (StataCorp. 2019. Stata Statistical Software: Release 16. College Station, TX: StataCorp LLC.<br>We used a random effect maximal likelihood estimation model using the Stata module Mataan

For manuscripts utilizing custom algorithms or software that are central to the research but not yet described in published literature, software must be made available to editors and reviewers. We strongly encourage code deposition in a community repository (e.g. GitHub). See the Nature Portfolio guidelines for submitting code & software for further information.

Data

Policy information about availability of data

All manuscripts must include a data availability statement. This statement should provide the following information, where applicable:

- Accession codes, unique identifiers, or web links for publicly available datasets
- A description of any restrictions on data availability
- For clinical datasets or third party data, please ensure that the statement adheres to our policy

: All de-identified data collected for the study will be made available to others, including related documents, available with publication. A copy of the paper with supplementary data will be available from the archive of the University of Bielefeld Library (Universität Bielefeld Bibliothek) where the data will be made available (publikationsdienste.ub@uni-bielefeld.de

March 2021

Human research participants

Policy information about studies involving human research participants and Sex and Gender in Research.

Reporting on sex and gender	The term "Sex" as the biological attribute to indicate male or female biology was used.
Population characteristics	Age, sex and treatment data were obtained from the published papers include in the meta-analysis.
Recruitment	Patients for analyses recruited according to the published data and selection criteria as detailed in manuscript
Ethics oversight	Not required as only previously published medical literature accessed.

Note that full information on the approval of the study protocol must also be provided in the manuscript.

Field-specific reporting

Please select the one below that is the best fit for your research. If you are not sure, read the appropriate sections before making your selection.

☒ Life sciences ☐ Behavioural & social sciences ☐ Ecological, evolutionary & environmental sciences

For a reference copy of the document with all sections, see nature.com/documents/nr-reporting-summary-flat.pdf

Life sciences study design

All studies must disclose on these points even when the disclosure is negative.

Sample size	Sample size not determined as the aim was to find and include as many studies with patients and data parameters that could be meta-analysed.
Data exclusions	Published studies fwere assessed or inclusion and exclusion criteria, with help from OS. Duplicates removed, then study abstracts and titles were checked through. All non-studies, conference reports, case reports with < 5 patients, reviews without new or relevant data, all non-English studies or reports (Swedish, Danish, German, Norwegian, Chinese, Russian, Dutch all removed).<br>We included only studies for which it was possible to calculate paired differences in the number of surgery or recurrences per month. We excluded study participants for which no pre-immunisation data were available or if patients were directly immunised at clinical diagnosis
Replication	PG (corresponding author) did the initial database searches, then replicated by OS (senior co-author and professional statistician).
Randomization	Randomization not relevant to our study, we had to find studies which gave pre- and post-vaccination recurrences (or surgeries as surrogate for recurrence) and time periods involved for their patients
Blinding	Not required as above.

Reporting for specific materials, systems and methods

We require information from authors about some types of materials, experimental systems and methods used in many studies. Here, indicate whether each material, system or method listed is relevant to your study. If you are not sure if a list item applies to your research, read the appropriate section before selecting a response.

Materials & experimental systems

n/a	Involved in the study	
☒	☐	Antibodies
☒	☐	Eukaryotic cell lines
☒	☐	Palaeontology and archaeology
☒	☐	Animals and other organisms
☒	☐	Clinical data
☒	☐	Dual use research of concern

Methods

n/a	Involved in the study	
☒	☐	ChIP-seq
☒	☐	Flow cytometry
☒	☐	MRI-based neuroimaging
